# Factors in Patient-Clinician Interactions That Influence the Decision-Making Process of Older Patients with Dementia

**DOI:** 10.31662/jmaj.2024-0260

**Published:** 2025-11-21

**Authors:** Yoshihisa Hirakawa, Kaoruko Aita, Tami Saito, Reiko Ishiyama, Sanae Takanashi, Chiho Shimada, Hisayuki Miura

**Affiliations:** 1 Department of Health Research and Innovation, Aichi Comprehensive Health Science Center, Aichi, Japan; 2 University of Tokyo Graduate School of Humanities and Sociology, Tokyo, Japan; 3 Department of Social Science, National Center for Geriatrics and Gerontology, Aichi, Japan; 4 International University of Health and Welfare Graduate School of Development of Care and Network, Tokyo, Japan; 5 Kobe Women’s University Graduate School of Nursing Doctoral Program, Kobe, Japan; 6 Faculty of Humanities and Social Science, Saku University, Nagano, Japan; 7 Clinical Research Center, Yushoukai Medical Corporation, Tokyo, Japan

**Keywords:** Japan, older patients, patient-clinician interaction, autonomy, dementia, rapport, advance care planning, decision-making

## Abstract

**Introduction:**

The importance of promoting the autonomy of people with dementia has been globally emphasized. Several studies have investigated factors that impede and facilitate their decision-making. However, few studies have explored these factors in light of their decision-making process. Therefore, this study aimed to determine factors in patient-clinician interactions that influence patient autonomy and participation in decision-making among Japanese patients with dementia.

**Methods:**

The authors adopted qualitative methods to understand the perceptions of health care professionals such as physicians, nurses, physical therapists, care managers, and social workers. Between January and March 2022, individual in-depth interviews were conducted online with 24 health care professionals with ample experience in primary palliative care for dementia. The topics were the support provided in patients’ decision-making, the support provided to proxy decision-makers, and the efforts undertaken for building a relationship between patients and families or among multidisciplinary teams. All interviews were audio-recorded electronically and transcribed verbatim. These data were synthesized and analyzed using content analysis.

**Results:**

Four main themes were derived that captured the factors that influence the decision-making process of Japanese patients with dementia in patient-clinician interactions: rapport, decision-making capability, provision of explanation, and presentation of options. The findings highlighted the importance of building rapport with patients through communication in the early stages of dementia, improving patients’ decision-making capability and protecting vulnerable patients who cannot make decisions independently, explaining each option’s risks and benefits in a comprehensible manner, and presenting a wide range of options to patients in everyday decision-making.

**Conclusions:**

Overall, the themes were in accordance with the process of informed consent. The findings also showed that clinicians must understand the barriers to obtaining informed consent that arise from patients’ cognitive impairment, decline, and fluctuations, in addition to cultural factors.

## Introduction

Dementia is a chronic syndrome in which cognitive function decreases beyond the deterioration that can be expected from biological aging. This accelerated longitudinal decrease of cognitive capacity leads to a progressive loss of autonomy in patients with dementia ^(1), (2)^. Because autonomy allows people to construct their lives on the basis of their values and personality, maintaining autonomy is important for a good quality of life for most people with dementia ^(3), (4)^. Moreover, studies show that a perceived lack of control is detrimental to physical and mental health, and when older people feel a sense of control over their actions, their health and well-being improve ^(5)^. Even if they are institutionalized, it is important for these patients to participate in their care. Enabling people with dementia living in long-term care facilities to participate in decision-making is central to their self-determination and feelings of worth. It also boosts their dignity and personhood ^(6), (7), (8)^. However, unfortunately, older adults living in nursing homes are restricted to some degree from fulfilling their wishes ^(9)^.

Thus, a better understanding of autonomy will contribute to achieving the goals of patient-centered care and ensuring compassionate, high-quality care that respects patients’ values ^(10)^. The implementation of person-centered care has been widely advocated globally in various health care settings ^(11)^. Various policies have been initiated recently for people with dementia and their families, such as the World Health Organization’s global action plan on the public health response to dementia ^(12)^. Nonetheless, health care professionals face difficulties in implementing person-centered care interventions and seek to improve the principles and practice of person-centered care ^(13), (14)^. Thus, identifying the factors that affect the autonomy of people with dementia can help improve not only their dignity and quality of life but also end-of-life care. Previous qualitative studies have found several barriers to delivering person-centered care interventions to older people. Some of these barriers are health care professionals’ lack of knowledge, communication skills, and holistic approaches, limited intention to use or implement such interventions, and the lack of organizational and managerial support ^(11), (15), (16)^.

By and large, supporting the autonomy of people with dementia has been globally underlined, and many studies have endeavored to identify the factors that affect the decision-making of these individuals. However, few studies have attempted to determine these factors on the basis of their decision-making process (which includes assessments, planning, and behavioral changes). Therefore, this study aimed to identify factors in patient-clinician interactions that influence patient autonomy and participation in decision-making among Japanese patients with dementia. The study determined four themes that encase factors in patient-clinician interactions that influence the autonomy and participation in decision-making of older patients with dementia.

## Materials and Methods

The authors adopted qualitative methods to understand the perceptions of health care professionals, such as physicians, nurses, physical therapists, care managers, and social workers, regarding the current state of autonomy and decision-making among patients with dementia.

### Data collection

The authors of this study discussed and created a topic guide for conducting individual in-depth interviews. The topics that were decided were the support provided in patients’ decision-making, the support provided to proxy decision-makers, and the efforts undertaken for building a relationship between patients and families or among multidisciplinary teams. Twenty-four health care professionals with ample experience in primary palliative care for dementia were recruited through convenience sampling among the authors’ acquaintances. Table 1 lists their characteristics. Written and verbal informed consent was obtained from all interviewees before conducting the interviews. All interviews were conducted online between January and March 2022 through Zoom, facilitated by the first author (YH), a geriatrician with ample experience in qualitative research. All interviews lasted for approximately an hour. Although clinical expertise contributed to the depth of engagement, potential bias due to role-based dynamics was carefully considered throughout the study design. Furthermore, the semi-structured interview guide was iteratively revised with input from interdisciplinary colleagues to avoid suggestive framing and ensure open-ended exploration.

**Table 1.** Characteristics of the Interviewees.

Participant number	Profession	Specialty	Sex	Age range	City	Setting	Experience in dementia care (in years)	Other relevant information
1	Physician	Emergency medicine	Female	40-44	Tokyo	Home	Unknown	Home care physician with abundant clinical experience in emergency departments
2	Physician	Geriatric medicine	Female	40-44	Tokyo	Hospital	8	University faculty member with abundant clinical experience in home care
3	Physician	Home care medicine	Male	40-44	Tokyo	Hospital	Unknown	Geriatrician with ample experience in hospital, facility, and home care
4	Physician	Palliative medicine	Male	55-59	Akita	Hospital	15	
5	Physician	Palliative medicine	Male	40-44	Miyagi	Hospital	10	University faculty member with ample experience in home care
6	Nurse	Palliative care/geriatric care	Female	55-59	Hokkaido	Hospital	Unknown	Works at a geriatric hospital
7	Nurse	Geriatric care	Female	45-49	Hokkaido	Home	3	Works at a home-visit nursing station affiliated with a hospital
8	Nurse	None	Female	50-54	Morioka	Hospital	30	Is certified as a care manager and social worker, and has experience in home-visit nursing care and community-based integrated care
9	Nurse	Palliative care	Female	50-54	Akita	Hospital	13	Works at an outpatient ward and room for community health
10	Nurse	Geriatric care	Female	55-59	Saitama	Home	25	Works at a home-visit nursing station affiliated with a group home for older people with dementia
11	Nurse	Geriatric care	Female	40-44	Aichi	Hospital	10	Works at a dementia care unit
12	Nurse	Dementia care	Female	40-44	Aichi	Hospital	8	Works at a dementia care unit
13	Nurse	Dementia care	Female	35-39	Aichi	Hospital	10	Works at a dementia care unit
14	Nurse	Dementia care/Geriatric care	Female	60-64	Hiroshima	Home	20	None
15	Nurse	Geriatric care	Female	40-44	Nagasaki	Hospital	20	Works at a room for community health
16	Physical therapist	None	Male	40-44	Miyagi	Home	6	None
17	Care manager	None	Female	45-49	Akita	Home	20	Chief care manager in charge of education
18	Care manager	None	Female	45-49	Miyagi	Home	15	Has work experience in long-term care facilities for older people with dementia
19	Care manager	Dementia care	Female	40-44	Miyagi	Home	18	Has five years of experience of working in a long-term care facility
20	Social worker	None	Female	60-64	Chiba	Home	10	Works at a community center
21	Social worker	None	Female	45-49	Nagano	Hospital	30	Works at a room for community health
22	Social worker	None	Female	40-44	Niigata	Home	10	Works at a home clinic providing palliative care
23	Social worker	None	Male	30-34	Gunma	Hospital	10	Works at a room for community health in a rehabilitation hospital
24	Social worker	None	Male	40-44	Kanagawa	Facility	10	Founder of small-scale long-term care facilities

### Data analysis

All interviews were audio-recorded electronically and transcribed verbatim. These data were synthesized and analyzed using content analysis ^(17)^, and ideas and patterns were identified systematically. First, the textual data were read multiple times to understand the overall content. Then, the data were arranged into several meaning units. All meaning units were then grouped into common meaning groups to identify larger themes. To enhance analytical neutrality, the grouping and content analysis process involved multiple discussions among all co-authors. These collaborative reviews allowed cross-validation of interpretations, identification of latent patterns, and refinement of emergent themes.

## Results

Four main themes were identified that captured factors in patient-clinician interactions that influence the decision-making process of Japanese patients with dementia. They were rapport, decision-making capability, explanation, and presentation of options.

### Rapport

Better communication can make it easier to meet the needs of patients with dementia. It can also help patients and clinicians understand each other. As the illness progresses, patients with dementia gradually lose their ability to communicate and find it increasingly difficult to express themselves clearly. Some participants were aware of the importance of communication in the early stages of dementia when most patients function independently and can engage in meaningful conversations and social activities.

“I believe that home-visit nursing care be initiated as early as possible to make sure they are involved in decision-making before a significant cognitive decline occurs” (participant number 14).

Communication difficulties in patients with dementia are a major source of stress for both clinicians and patients’ families. Consequently, clinicians discuss patient care and interventions with family members and want them to be the decision-makers, not the patient. In such cases, some participants tried not to show this to the patients.

“When I want to talk to their family caregivers, I try to do it outside the house so that patients with dementia don’t find out” (participant number 2).

Patients with dementia often hate their situation and loss of independence, which makes them resist clinicians’ caregiving. Some participants emphasized the importance of treating patients nicely before starting a conversation.

“I think that healthcare professionals should bring down the patient’s guard before initiating a conversation with them” (participant number 20).

### Decision-making capability

Dementia alters an individual’s decision-making capability. Moreover, there is a common misconception that people with dementia are incapable of making decisions. However, individuals with moderate dementia differ in their ability to participate in decision-making.

“I do not think Yes/No questions are good for assessing how capable a patient with dementia is of making decisions. Instead, clinicians should perform a cognitive assessment that has scale-based questions” (participant number 21).

“Fluctuations in the cognitive function of patients with dementia prevent healthcare professionals from determining the central axis of decision-making” (participant number 16).

### Explanation

Having informed consent means that the patient knows all possible consequences of having a medical treatment or procedure, including the risks, benefits, alternative treatments, and potential side effects, and consents to undergo it. To have this consent, health care professionals must provide adequate information and options so that individuals can make informed decisions. Some participants believed that obtaining informed consent was challenging because it is difficult to understand the meaning of treatment and options, especially for older people with dementia.

“Healthcare professionals should use easier words and phrases so that patients with dementia and their family caregivers can understand the details of medical treatment” (participant number 9).

“Healthcare professionals should carefully explain why they don’t recommend an option the client has chosen” (participant number 5).

### Presentation of options

Clinicians emphasize ways autonomy and involvement in decision-making can help enhance the quality of life of older patients with dementia. Such options include palliative care, do-not-resuscitate orders, refusal or withdrawal of treatment, and refusal of food and drink. In a broader sense, everyday decision-making encompasses decisions concerning daily activities, such as what to eat, what to do, and what to wear. However, some participants stated that older patients with dementia are often excluded from decision-making, even when it is related to daily functioning.

“Before they discuss end-of-life care, healthcare professionals must discuss daily-life decisions with their clients” (participant number 14).

The purpose of decision-making discussion also includes the provision of spiritual care. Participants recognized the importance of executing patients’ wishes as much as possible, even when patients are nearing death.

“Since recognizing dementia as a life-limiting condition benefits patients with it, I am always thinking about when I should start to prioritize the bucket list over treatment” (participant number 6).

## Discussion

This study revealed factors in patient-clinician interactions that influence the decision-making process of older patients with dementia. Four themes encapsulate these factors: rapport between the two parties, the decision-making capability of patients, the explanation provided by health care professionals, and the presentation of options. These themes were eventually in accordance with the process of informed consent. The findings also showed that clinicians must understand and respond to the challenges of obtaining informed consent that arise from cognitive impairment, decline, and fluctuations.

The results of this study emphasized the importance of building rapport through communication in the early stages of dementia. Rapport is considered fundamental to building clinical relationships. However, the theoretical background and key concepts of building rapport with patients with dementia remain scant ^(18)^. Moreover, health care professionals’ negative attitudes toward patients with dementia have been well documented ^(19), (20), (21), (22)^, and these attitudes may affect communication between patients and health care providers. Previous studies have suggested reminiscence and life review as vital elements in rapport building. They can help health care professionals understand patients’ everyday experiences, ideas, concerns, and expectations more effectively ^(23), (24), (25)^. When it comes to decision-making regarding end-of-life care, advance care planning (ACP) delivers person-centered end-of-life care based on the individual’s wishes ^(26)^. However, previous studies have noted that patients’ non-medical needs, such as values and personal preferences, are likely to be omitted from ACP discussions because of a lack of awareness on the part of health care professionals ^(26), (27)^. Thus, educational support must be provided to health care professionals so that they acquire the communication skills needed to build rapport with patients with dementia, from a person-centered perspective.

The results showed that health care professionals should improve patients’ decision-making capacity and protect vulnerable patients who cannot make decisions independently because of dementia. The decision-making of people with dementia or severe mental illness has attracted widespread attention ^(28), (29), (30)^. A wide range of instruments are available for assessing mental capacity. However, no standardized assessment exists for patients with dementia that can encourage the adoption and operationalization of recommended practices ^(31)^. The results also showed that clinicians focus on fluctuating cognition when assessing patients’ decision-making capability. Fluctuating cognition is a feature of dementia, especially in patients with Lewy bodies; however, it is challenging to assess ^(32)^. One study evaluated the reliability and validity of the Clinician Assessment of Fluctuation (CAF), which assesses fluctuating cognition in patients with dementia ^(33)^. Another study evaluated the value of the One Day Fluctuation Assessment Scale (ODFAS) and the CAF and found that both scales are useful for the clinical assessment of fluctuation in dementia ^(34)^.

The findings revealed that clinicians should convey all relevant information to patients with dementia in a way they can understand to obtain informed consent. Regardless of cognitive function, explaining end-of-life care and treatment options to older people remains challenging. This is partly because complex medical terms, concepts, and uncertainties can create a barrier between clinicians and patients ^(35), (36), (37)^. Moreover, patients’ understanding of such information depends on their education level and personality ^(36)^. Older patients with dementia often require family members and others to assume decision-making responsibilities. Despite decades of debate, policies regulating the decision-making processes of patients with dementia are not well defined. Policy discussions and future research should consider that building congruence between families’ decisions and patients’ values can mitigate ethical reservations about involving patients who are incapacitated in decision-making ^(38)^. Recent studies have focused on shared decision-making as a means of allowing families, people with dementia, and even health care professionals to make choices, be autonomous, and participate in end-of-life care ^(39), (40)^.

Finally, recent findings underscore the importance of offering a wide range of everyday decision-making options to patients with dementia, emphasizing that care should be grounded in the individual’s daily wishes, particularly for those living at home ^(41)^. Despite evidence that many patients―whether in institutions or community settings―can participate in everyday decisions, they are often denied the opportunity ^(42)^. Health care professionals are therefore urged to enhance patients’ decision-making capacity while safeguarding those unable to decide independently owing to cognitive decline ^(28), (30)^. Although numerous tools exist to assess mental capacity, there remains no standardized method tailored to dementia that facilitates the adoption of best practices ^(31)^. Clinicians often focus on fluctuating cognition, a hallmark of dementia―especially Lewy body dementia―yet its assessment remains complex ^(32)^. Instruments such as the CAF and the ODFAS have indicated clinical utility in evaluating these fluctuations ^(33), (34)^. In Japan, cultural norms favor family-centered and paternalistic decision-making, which can both support and constrain patient autonomy ^(43)^. Family members frequently act as surrogate decision-makers, sometimes prioritizing collective harmony over the patient’s expressed preferences. Although this may alleviate emotional burden, it risks marginalizing the patient’s voice. Clinicians must therefore navigate these relational dynamics with cultural sensitivity, ensuring that ethical principles such as respect for autonomy are upheld within culturally embedded practices ^(28), (29), (30)^.

This study has some limitations. First, although several health care professionals were recruited, the sample size was relatively small. Future qualitative studies may yield a wider range of perspectives and information by having a larger sample. Second, patients with dementia and their families were not included as study participants. This was because the research team took coronavirus disease 2019 (COVID-19)-induced social distancing measures into account. Owing to COVID-19-related institutional restrictions, it was not feasible to include interviews with patients and their families in this study. This limitation caused the absence of essential perspectives regarding patients’ subjective experiences and emotional responses in the decision-making process. Consequently, the interpretation of themes may reflect a professional-centric lens shaped primarily by clinicians’ viewpoints. Nonetheless, the primary objective of this study was to elucidate the structural support mechanisms used by health care professionals in clinical contexts, which aligns with the chosen participant pool. As a future direction, incorporating qualitative data from patients and family members would allow a more holistic understanding of bidirectional decision-making and relational dynamics. This approach could further inform dementia care practices centered on autonomy support and the development of relational infrastructure. Third, in Japan, decision-making involves strong family involvement and paternalism. Owing to this cultural aspect ^(43)^, the findings of this study may not be generalizable to other countries. Finally, given the first author, a physician, was the only interviewer, data collected from individual interviews may have been susceptible to social-desirability bias.

### Conclusions

This study aimed to identify factors in patient-clinician interactions that influence patient autonomy and participation in decision-making among Japanese patients with dementia. The results highlighted the importance of building rapport, assessing and improving patients’ decision-making capability, explaining each option’s risks and benefits in a comprehensible manner, and presenting a wide range of options. These four themes were contained in the basic elements of informed consent. Furthermore, clinicians should consider the challenges in obtaining informed consent that stem from cultural features and patients’ cognitive impairment, decline, and fluctuations.

## Article Information

### Acknowledgments

The authors thank all participants for the time and energy they devoted to this study.

### Author Contributions

Conceptualization, Yoshihisa Hirakawa, Kaoruko Aita, Tami Saito, Reiko Ishiyma, Sanae Takanashi, Chiho Shimada, and Hisayuki Miura; Methods, Yoshihisa Hirakawa, Kaoruko Aita, and Hisayuki Miura; Formal analysis, Yoshihisa Hirakawa, Kaoruko Aita, and Hisayuki Miura; Investigation, Yoshihisa Hirakawa, Kaoruko Aita, Tami Saito, Reiko Ishiyma, Sanae Takanashi, Chiho Shimada, and Hisayuki Miura ; Data curation, Yoshihisa Hirakawa; Writing―original draft preparation, Yoshihisa Hirakawa; Writing―review and editing, Yoshihisa Hirakawa, Kaoruko Aita, Tami Saito, Reiko Ishiyma, Sanae Takanashi, Chiho Shimada, and Hisayuki Miura; Supervision, Kaoruko Aita and Hisayuki Miura; Funding acquisition, Hisayuki Miura.

### Conflicts of Interest

None

### IRB Approval Code and Name of the Institution

This study was reviewed and approved by the Bioethics Review Committee of the Nagoya University School of Medicine, Japan (approval No. 2015-0444).

## References

[1] Hegde S, Ellajosyula R. Capacity issues and decision-making in dementia. Ann Indian Acad Neurol. 2016;19(suppl 1):S34-9. 27891023 10.4103/0972-2327.192890 PMC5109759

[2] Bentwich ME, Dickman N, Oberman A. Autonomy and dignity of patients with dementia: perceptions of multicultural caretakers. Nurs Ethics. 2018;25(1):37-53. 27113259 10.1177/0969733016642625

[3] Piers R, Albers G, Gilissen J, et al. Advance care planning in dementia: recommendations for healthcare professionals. BMC Palliat Care. 2018;17(1):88. 29933758 10.1186/s12904-018-0332-2 PMC6014017

[4] Bosisio F, Sterie AC, Rubli Truchard E, et al. Implementing advance care planning in early dementia care: results and insights from a pilot interventional trial. BMC Geriatr. 2021;21(1):573. 34666711 10.1186/s12877-021-02529-8 PMC8524211

[5] Andresen M, Puggaard L. Autonomy among physically frail older people in nursing home settings: a study protocol for an intervention study. BMC Geriatr. 2008;8:32. 19040767 10.1186/1471-2318-8-32 PMC2631025

[6] Wulff I, Kölzsch M, Kalinowski S, et al. Perceived enactment of autonomy of nursing home residents: a German cross-sectional study. Nurs Health Sci. 2013;15(2):186-93. 23210863 10.1111/nhs.12016

[7] Beattie E, O’Reilly M, Fetherstonhaugh D, et al. Supporting autonomy of nursing home residents with dementia in the informed consent process. Dementia (London). 2019;18(7-8):2821-35. 29512408 10.1177/1471301218761240

[8] Van Loon J, Luijkx K, Janssen M, et al. Facilitators and barriers to autonomy: a systematic literature review for older adults with physical impairments, living in residential care facilities. Ageing Soc. 2021;41(5):1021-50.

[9] Boumans J, van Boekel LC, Baan CA, et al. How can autonomy be maintained and informal care improved for people with dementia living in residential care facilities: a systematic literature review. Gerontologist. 2019;59(6):e709-30. 30239712 10.1093/geront/gny096 PMC6858830

[10] Mapes MV, Depergola PA, McGee WT. Patient-centered care and autonomy: shared decision-making in practice and a suggestion for practical application in the critically ill. J Intensive Care Med. 2020;35(11):1352-5. 31451000 10.1177/0885066619870458

[11] Moore L, Britten N, Lydahl D, et al. Barriers and facilitators to the implementation of person-centred care in different healthcare contexts. Scand J Caring Sci. 2017;31(4):662-73. 27859459 10.1111/scs.12376 PMC5724704

[12] Cahill S. WHO’s global action plan on the public health response to dementia: some challenges and opportunities. Aging Ment Health. 2020;24(2):197-9. 30600688 10.1080/13607863.2018.1544213

[13] Kloos N, Drossaert CHC, Trompetter HR, et al. Exploring facilitators and barriers to using a person centered care intervention in a nursing home setting. Geriatr Nurs. 2020;41(6):730-9. 32460962 10.1016/j.gerinurse.2020.04.018

[14] Riding S, Glendening N, Heaslip V. Real world challenges in delivering person-centred care: a community-based case study. Br J Community Nurs. 2017;22(8):391-6. 28767302 10.12968/bjcn.2017.22.8.391

[15] Younas A, Inayat S, Masih S. Nurses’ perceived barriers to the delivery of person-centred care to complex patients: a qualitative study using theoretical domains framework. J Clin Nurs. 2023;32(3-4):368-81. 35132737 10.1111/jocn.16245

[16] Oppert ML, O’Keeffe VJ, Duong D. Knowledge, facilitators and barriers to the practice of person-centred care in aged care workers: a qualitative study. Geriatr Nurs. 2018;39(6):683-8. 29859699 10.1016/j.gerinurse.2018.05.004

[17] Elo S, Kääriäinen M, Kanste O, et al. Qualitative content analysis: a focus on trustworthiness. Sage Open. 2014;4(1):2158244014522633.

[18] English W, Gott M, Robinson J. The meaning of rapport for patients, families, and healthcare professionals: a scoping review. Patient Educ Couns. 2022;105(1):2-14. 34154861 10.1016/j.pec.2021.06.003

[19] Zhao W, Moyle W, Wu MW, et al. Hospital healthcare professionals’ knowledge of dementia and attitudes towards dementia care: a cross-sectional study. J Clin Nurs. 2022;31(13-14):1786-99. 33295010 10.1111/jocn.15590

[20] Gove D, Downs M, Vernooij-Dassen M, et al. Stigma and GPs’ perceptions of dementia. Aging Ment Health. 2016;20(4):391-400. 25765096 10.1080/13607863.2015.1015962

[21] Evripidou M, Charalambous A, Middleton N, et al. Nurses’ knowledge and attitudes about dementia care: systematic literature review. Perspect Psychiatr Care. 2019;55(1):48-60. 29766513 10.1111/ppc.12291

[22] Gerritsen DL, van Beek APA, Woods RT. Relationship of care staff attitudes with social well-being and challenging behavior of nursing home residents with dementia: a cross sectional study. Aging Ment Health. 2019;23(11):1517-23. 30409022 10.1080/13607863.2018.1506737

[23] Doran C, Noonan M, Doody O. Life-story work in long-term care facilities for older people: an integrative review. J Clin Nurs. 2019;28(7-8):1070-84. 30431682 10.1111/jocn.14718

[24] Subramaniam P, Woods B, Whitaker C. Life review and life story books for people with mild to moderate dementia: a randomised controlled trial. Aging Ment Health. 2014;18(3):363-75. 24063317 10.1080/13607863.2013.837144 PMC4017276

[25] de Vries K. Communicating with older people with dementia. Nurs Older People. 2013;25(4):30-7. 10.7748/nop2013.05.25.4.30.e429 23789241

[26] Muraya T, Akagawa Y, Andoh H, et al. Improving person-centered advance care planning conversation with older people: a qualitative study of core components perceived by healthcare professionals. J Rural Med. 2021;16(4):222-8. 34707731 10.2185/jrm.2021-022 PMC8527630

[27] Rosa WE, Izumi S, Sullivan DR, et al. Advance care planning in serious illness: a narrative review. J Pain Symptom Manag. 2023;65(1):e63-e78. 10.1016/j.jpainsymman.2022.08.012 PMC9884468 36028176

[28] Darby RR, Dickerson BC. Dementia, decision making, and capacity. Harv Rev Psychiatry. 2017;25(6):270-8. 29117022 10.1097/HRP.0000000000000163 PMC5711478

[29] Aoki Y. Shared decision making for adults with severe mental illness: a concept analysis. Jpn J Nurs Sci. 2020;17(4):e12365. 32761783 10.1111/jjns.12365 PMC7590107

[30] Mountford W, Dening KH, Green J. Advance care planning and decision-making in dementia care: a literature review. Nurs Older People. 2020. 10.7748/nop.2020.e1238 32666719

[31] Pennington C, Davey K, Ter Meulen R, et al. Tools for testing decision-making capacity in dementia. Age Ageing. 2018;47(6):778-84. 30010696 10.1093/ageing/afy096

[32] Shulman KI, Hull IM, Dekoven S, et al. Cognitive fluctuations and the lucid interval in dementia: implications for testamentary capacity. J Am Acad Psychiatry Law. 2015;43(3):287-92. 26438805

[33] Van Dyk K, Towns S, Tatarina O, et al. Assessing fluctuating cognition in dementia diagnosis: interrater reliability of the clinician assessment of fluctuation. Am J Alzheimers Dis Other Demen. 2016;31(2):137-43. 26340964 10.1177/1533317515603359 PMC4758876

[34] Walker MP, Ayre GA, Cummings JL, et al. The clinician assessment of fluctuation and the one day fluctuation assessment scale. Two methods to assess fluctuating confusion in dementia. Br J Psychiatry. 2000;177:252-6. 11040887 10.1192/bjp.177.3.252

[35] Hirakawa Y, Chiang C, Yasuda Uemura M, et al. Involvement of Japanese care managers and social workers in advance care planning. J Soc Work End Life Palliat Care. 2018;14(4):315-27. 30653395 10.1080/15524256.2018.1533912

[36] Giampieri M. Communication and informed consent in elderly people. Minerva Anestesiol. 2012;78(2):236-42. 22127308

[37] Khizar B, Harwood RH. Making difficult decisions with older patients on medical wards. Clin Med (Lond). 2017;17(4):353-6. 28765415 10.7861/clinmedicine.17-4-353 PMC6297638

[38] Kim SYH. The ethics of informed consent in Alzheimer disease research. Nat Rev Neurol. 2011;7(7):410-4. 21610690 10.1038/nrneurol.2011.76 PMC3475518

[39] Miller LM, Whitlatch CJ, Lyons KS. Shared decision-making in dementia: a review of patient and family carer involvement. Dementia (London). 2016;15(5):1141-57. 25370075 10.1177/1471301214555542

[40] Mattos MK, Gibson JS, Wilson D, et al. Shared decision-making in persons living with dementia: a scoping review. Dementia (London). 2023;22(4):875-909. 36802973 10.1177/14713012231156976 PMC10866150

[41] Mamun MR, Hirakawa Y, Saif-Ur-Rahman KM, et al. Everyday wishes of older people living with dementia in care planning: a qualitative study. BMC Health Serv Res. 2022;22(1):184. 35151313 10.1186/s12913-022-07606-1 PMC8840703

[42] Smebye KL, Kirkevold M, Engedal K. How do persons with dementia participate in decision making related to health and daily care? A multi-case study. BMC Health Serv Res. 2012;12:241. 22870952 10.1186/1472-6963-12-241 PMC3475075

[43] Akabayashi A, Nakazawa E. Autonomy in Japan: what does it look like? Asian Bioeth Rev. 2022;14(4):317-36. 36203709 10.1007/s41649-022-00213-6 PMC9530074

